# *N*,*N*′-Dibenzyl-*N*,*N*′-dimethyl-*N*′′-(2-phenyl­acet­yl)phospho­ric triamide

**DOI:** 10.1107/S1600536811049178

**Published:** 2011-11-25

**Authors:** Mehrdad Pourayoubi, Samad Shoghpour, Laura Torre-Fernández, Santiago García-Granda

**Affiliations:** a Department of Chemistry, Ferdowsi University of Mashhad, Mashhad 91779, Iran; b Departamento de Química Física y Analítica, Facultad de Química, Universidad de Oviedo–CINN, C/ Julián Clavería, 8, 33006 Oviedo, Asturias, Spain

## Abstract

The P atom in the title mol­ecule, C_24_H_28_N_3_O_2_P, is in a distorted tetra­hedral P(=O)(N)(N)_2_ environment. The phosphoryl group and the NH unit adopt a *syn* orientation with respect to each other and the N atoms have *sp* ^2^ character. The P—N bonds in the P(O)[N(CH_3_)(CH_2_C_6_H_5_)]_2_ unit are shorter than the P—N bond in the C(=O)NHP(=O) fragment. An intra­molecular C—H⋯O hydrogen bond occurs. In the crystal, pairs of P=O⋯H—N hydrogen bonds form centrosymmetric dimers. C—H⋯O contacts are also observed. Four C atoms of two benzene rings are disordered over two alternative sites with an occupancy ratio of 0.523 (12):0.427 (12).

## Related literature

For hydrogen-bond patterns in compounds with formula *R*C(O)NHP(O)[N*R* ^1^ *R* ^2^]_2_ and *R*C(O)NHP(O)[NH*R* ^1^]_2_, see: Toghraee *et al.* (2011 ▶). For hydrogen-bond strengths and for bond lengths and angles in a related structure, see: Pourayoubi *et al.* (2011 ▶). 
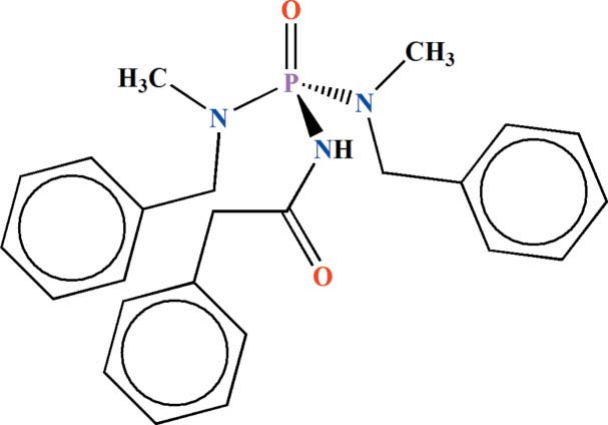


## Experimental

### 

#### Crystal data

C_24_H_28_N_3_O_2_P
*M* *_r_* = 421.46
Monoclinic, 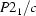

*a* = 12.4823 (4) Å
*b* = 10.3535 (3) Å
*c* = 20.0392 (5) Å
β = 118.646 (3)°
*V* = 2272.78 (13) Å^3^
*Z* = 4
Cu *K*α radiation
μ = 1.26 mm^−1^
*T* = 120 K
0.21 × 0.08 × 0.04 mm

#### Data collection

Agilent Xcalibur Gemini R diffractometer
Absorption correction: multi-scan (*CrysAlis PRO*; Agilent, 2011 ▶) *T*_min_ = 0.852, *T*_max_ = 1.000
10604 measured reflections
4244 independent reflections
3366 reflections with *I* > 2σ(*I*)
*R*_int_ = 0.034

#### Refinement

*R*[*F* ^2^ > 2σ(*F* ^2^)] = 0.073
*wR*(*F* ^2^) = 0.216
*S* = 1.07
4244 reflections
287 parameters
13 restraints
H atoms treated by a mixture of independent and constrained refinement
Δρ_max_ = 1.30 e Å^−3^
Δρ_min_ = −0.46 e Å^−3^

### 

Data collection: *CrysAlis PRO* (Agilent, 2011 ▶); cell refinement: *CrysAlis PRO*; data reduction: *CrysAlis PRO*; program(s) used to solve structure: *SIR92* (Altomare *et al.*, 1994 ▶); program(s) used to refine structure: *SHELXL97* (Sheldrick, 2008 ▶); molecular graphics: *Mercury* (Macrae *et al.*, 2008 ▶) and *ORTEP-3* (Farrugia, 1997 ▶); software used to prepare material for publication: *WinGX* (Farrugia, 1999 ▶) and *enCIFer* (Allen *et al.*, 2004 ▶).

## Supplementary Material

Crystal structure: contains datablock(s) I, global. DOI: 10.1107/S1600536811049178/fy2027sup1.cif

Structure factors: contains datablock(s) I. DOI: 10.1107/S1600536811049178/fy2027Isup2.hkl

Additional supplementary materials:  crystallographic information; 3D view; checkCIF report

## Figures and Tables

**Table 1:** Hydrogen-bond geometry (Å, °)

*D*—H⋯*A*	*D*—H	H⋯*A*	*D*⋯*A*	*D*—H⋯*A*
C25—H25*A*⋯O3	0.97	2.49	3.347 (5)	147
N5—H5⋯O2^i^	0.86	1.95	2.763 (3)	156
C28—H28*A*⋯O2^i^	0.97	2.57	3.351 (4)	138
C17—H17⋯O2^ii^	0.93	2.51	3.443 (5)	176
C28—H28*B*⋯O3^iii^	0.97	2.40	3.325 (4)	160

Symmetry codes: (i) 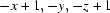; (ii) 
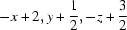
; (iii) 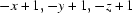.

**Fig. 1.**
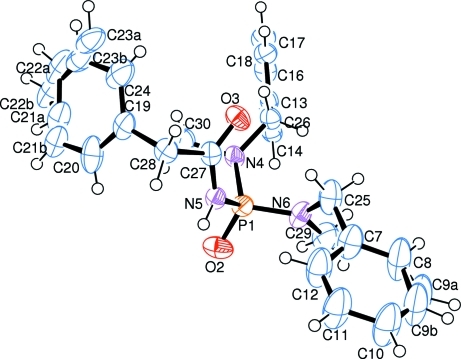
The molecular structure of the title compound with ellipsoids shown at the 50% probability level.

**Fig. 2.**
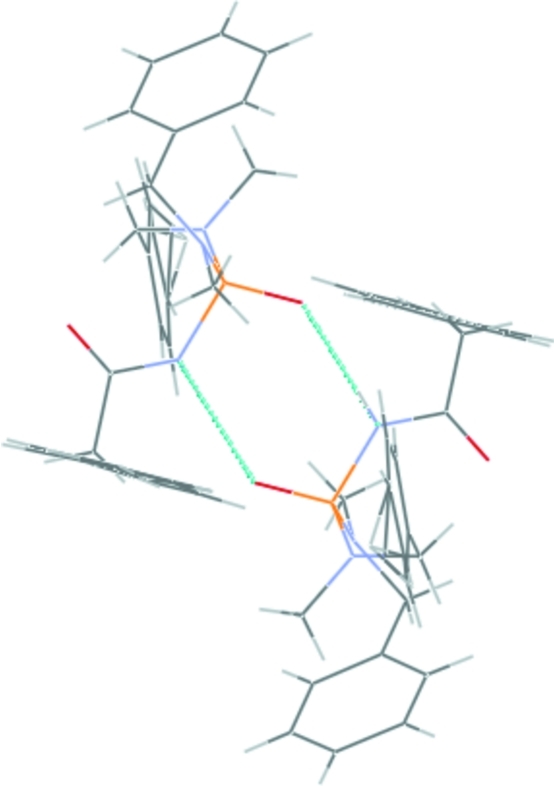
A view of the centrosymmetric dimer formed by H-bonding.

## supplementary crystallographic information

### Comment

The hydrogen bond patterns and strengths in two subclasses of acetyl phosphoric triamide compounds with formula RC(O)NHP(O)[N*R*^1^*R*^2^]_2_ and RC(O)NHP(O)[NH*R*^1^]_2_ were analyzed, respectively, by Toghraee *et al.* (2011) and by Pourayoubi *et al.* (2011).

The structure determination of the title molecule, C_6_H_5_CH_2_C(O)N(H)P(O)[N(CH_3_)(CH_2_C_6_H_5_)]_2_ (Fig. 1), was performed because of our interest in the structural characteristics of new compounds with a C(═O)NHP(═O)(N)_2_ skeleton, which belong to the phosphoric triamide family.

Single crystals of the title molecule were obtained from CH_3_CN after slow evaporation at room temperature. The P atom is placed in a distorted tetrahedral P(═O)(N)(N)_2_ environment with the surrounding bond angles in the range of 106.38 (13)°-112.48 (17)°. The P—N bond in the C(O)NHP(O) moiety (with length of 1.681 (3) Å) is longer than the two other P—N bonds (1.621 (3) Å & 1.633 (3) Å). The P═O bond length is standard for this family of phosphoramidate compounds (Pourayoubi *et al.*, 2011).

The angles at the tertiary N atoms confirm their *sp*^2^ character. Moreover, the C—N—P angle in the C(O)NHP(O) fragment is 126.3 (2)°.

The hydrogen atom of the C(═O)NHP(═O) group is involved in an intermolecular –P═O···H—N– hydrogen bond (see Table 1). A pair of this hydrogen bond forms a centrosymmetric dimer, see Figure 2, which is a usual H-bond pattern for compounds of the formula RC(O)NHP(O)[NR'*R*"]_2_, where *R*' and *R*" ≠ H, and in the case of a *syn* orientation of P═O *versus* N—H (Toghraee *et al.*, 2011).

### Experimental

Reaction of phosphorus pentachloride (1.85 mmol) and 2-phenylacetamide (1.85 mmol) in dry CCl_4_ (15 ml) at 353 K (3 h) followed by treatment with formic acid (1.85 mmol) at room temperature leads to the formation of C_6_H_5_CH_2_C(O)NHP(O)Cl_2_ as a solid-oily product (stage I). A solution of *N*-methylbenzylamine (7.4 mmol) in CHCl_3_ (5 ml) was added dropwise to a solution containing the total product of stage I in CHCl_3_ (15 ml) at 273 K. After 6 h of stirring, the solvent was evaporated in vacuum. The obtained solid was washed with distilled water. Single crystals were obtained from a solution of the title compound in CH_3_CN after slow evaporation at room temperature. The crystals were washed with CCl_4_ to remove the oily layer from the surface of the crystals.

### Refinement

At the end of the refinement the highest peak in the electron density was 1.300 e Å ^-3^, while the deepest hole was -0.460 e Å ^-3^. In order to refine the disorder shown by the C9, C21, C22 and C23 atoms, EADP restraints were used and the distances C23A–C21A, C21A–C22A, C22A–C23A, C9A–H9A, C23A–H23A and C22A–H23A had to be fixed. Flat group restraints were used to fix the geometry of the atoms labeled with A, i.e., those belonging to the minor disorder component. The occupancy of these atoms refined to 0.427 (12). H atoms labeled H9A, H21A and H23A were located in a difference map and were allowed to ride on the parent atom with *U*_iso_(H) = 1.2 *U*_eq_(C). The rest of the H atoms were geometrically placed and refined in riding mode with isotropic displacements calculated from the *U*_eq_ of the parent atom.

### Crystal data

**Table:**

C_24_H_28_N_3_O_2_P	*F*(000) = 896
*M**_r_* = 421.46	*D*_x_ = 1.232 Mg m^−^^3^
Monoclinic, *P*2_1_/*c*	Cu *K*α radiation, λ = 1.54180 Å
Hall symbol: -P 2ybc	Cell parameters from 4446 reflections
*a* = 12.4823 (4) Å	θ = 3.6–70.4°
*b* = 10.3535 (3) Å	µ = 1.26 mm^−^^1^
*c* = 20.0392 (5) Å	*T* = 120 K
β = 118.646 (3)°	Prismatic, colourless
*V* = 2272.78 (13) Å^3^	0.21 × 0.08 × 0.04 mm
*Z* = 4	

### Data collection

**Table:**

Agilent Xcalibur Gemini R diffractometer	4244 independent reflections
Radiation source: Enhance (Cu) X-ray Source	3366 reflections with *I* > 2σ(*I*)
graphite	*R*_int_ = 0.034
Detector resolution: 10.2673 pixels mm^-1^	θ_max_ = 70.6°, θ_min_ = 4.0°
ω scans	*h* = −15→12
Absorption correction: multi-scan (*CrysAlis PRO*; Agilent, 2011)	*k* = −8→12
*T*_min_ = 0.852, *T*_max_ = 1.000	*l* = −21→24
10604 measured reflections	

### Refinement

**Table:**

Refinement on *F*^2^	Primary atom site location: structure-invariant direct methods
Least-squares matrix: full	Secondary atom site location: difference Fourier map
*R*[*F*^2^ > 2σ(*F*^2^)] = 0.073	Hydrogen site location: inferred from neighbouring sites
*wR*(*F*^2^) = 0.216	H atoms treated by a mixture of independent and constrained refinement
*S* = 1.07	*w* = 1/[σ^2^(*F*_o_^2^) + (0.117*P*)^2^ + 2.5265*P*] where *P* = (*F*_o_^2^ + 2*F*_c_^2^)/3
4244 reflections	(Δ/σ)_max_ < 0.001
287 parameters	Δρ_max_ = 1.30 e Å^−^^3^
13 restraints	Δρ_min_ = −0.46 e Å^−^^3^

### Special details

**Table:**

Geometry. All e.s.d.'s (except the e.s.d. in the dihedral angle between two l.s. planes) are estimated using the full covariance matrix. The cell e.s.d.'s are taken into account individually in the estimation of e.s.d.'s in distances, angles and torsion angles; correlations between e.s.d.'s in cell parameters are only used when they are defined by crystal symmetry. An approximate (isotropic) treatment of cell e.s.d.'s is used for estimating e.s.d.'s involving l.s. planes.
Refinement. Refinement of *F*^2^ against ALL reflections. The weighted *R*-factor *wR* and goodness of fit *S* are based on *F*^2^, conventional *R*-factors *R* are based on *F*, with *F* set to zero for negative *F*^2^. The threshold expression of *F*^2^ > σ(*F*^2^) is used only for calculating *R*-factors(gt) *etc*. and is not relevant to the choice of reflections for refinement. *R*-factors based on *F*^2^ are statistically about twice as large as those based on *F*, and *R*- factors based on ALL data will be even larger.

### Fractional atomic coordinates and isotropic or equivalent isotropic displacement parameters (Å^2^)

**Table:**

	*x*	*y*	*z*	*U*_iso_*/*U*_eq_	Occ. (<1)
P1	0.61467 (7)	0.09793 (8)	0.61667 (4)	0.0349 (3)	
O2	0.6047 (2)	−0.0406 (2)	0.59532 (13)	0.0495 (7)	
O3	0.6162 (2)	0.3822 (2)	0.57253 (13)	0.0433 (6)	
N4	0.7567 (2)	0.1457 (3)	0.65817 (14)	0.0348 (6)	
N5	0.5375 (2)	0.1807 (3)	0.53507 (14)	0.0332 (6)	
H5	0.4775	0.1415	0.4982	0.040*	
N6	0.5563 (2)	0.1285 (3)	0.67185 (15)	0.0454 (8)	
C7	0.3578 (4)	0.2146 (5)	0.6449 (2)	0.0633 (12)	
C8	0.3112 (5)	0.2069 (7)	0.6953 (3)	0.0907 (15)	
H8	0.3664	0.2167	0.7467	0.109*	
C9A	0.1977 (19)	0.1873 (19)	0.6773 (11)	0.0907 (15)	0.427 (12)
C9B	0.1788 (14)	0.1750 (18)	0.6644 (8)	0.0907 (15)	0.573 (12)
H9B	0.1479	0.1622	0.6979	0.109*	0.573 (12)
C10	0.1089 (4)	0.1652 (7)	0.5943 (3)	0.092 (2)	
H10	0.0252	0.1561	0.5757	0.110*	
C11	0.1562 (4)	0.1681 (7)	0.5449 (3)	0.0878 (19)	
H11	0.1045	0.1539	0.4934	0.105*	
C12	0.2785 (4)	0.1914 (7)	0.5696 (3)	0.0835 (18)	
H12	0.3075	0.1914	0.5347	0.100*	
C13	0.9382 (3)	0.2014 (3)	0.77852 (16)	0.0331 (7)	
C14	0.9627 (3)	0.0783 (3)	0.80961 (18)	0.0385 (7)	
H14	0.8996	0.0188	0.7954	0.046*	
C15	1.0808 (3)	0.0436 (4)	0.86178 (19)	0.0463 (9)	
H15	1.0964	−0.0390	0.8825	0.056*	
C16	1.1755 (3)	0.1300 (4)	0.88326 (19)	0.0486 (9)	
H16	1.2548	0.1056	0.9178	0.058*	
C17	1.1521 (3)	0.2529 (4)	0.85324 (19)	0.0497 (9)	
H17	1.2154	0.3121	0.8677	0.060*	
C18	1.0334 (3)	0.2883 (4)	0.80124 (18)	0.0402 (8)	
H18	1.0179	0.3716	0.7815	0.048*	
C19	0.6284 (3)	0.3206 (4)	0.42633 (17)	0.0441 (9)	
C20	0.6540 (4)	0.2006 (5)	0.4061 (2)	0.0624 (12)	
H20	0.5970	0.1367	0.3988	0.075*	
C21A	0.7823 (16)	0.2127 (17)	0.4086 (9)	0.054 (3)	0.427 (12)
C21B	0.7447 (9)	0.1624 (14)	0.3957 (6)	0.054 (3)	0.573 (12)
H21B	0.7512	0.0794	0.3802	0.065*	0.573 (12)
C22A	0.8510 (17)	0.3232 (19)	0.4241 (9)	0.068 (4)	0.427 (12)
H22A	0.9253	0.3190	0.4240	0.081*	0.427 (12)
C22B	0.8303 (12)	0.2587 (18)	0.4101 (6)	0.068 (4)	0.573 (12)
H22B	0.8991	0.2415	0.4051	0.081*	0.573 (12)
C23A	0.815 (2)	0.4403 (17)	0.4398 (13)	0.068 (4)	0.427 (12)
C23B	0.8133 (15)	0.3803 (14)	0.4321 (9)	0.068 (4)	0.573 (12)
H23B	0.8743	0.4410	0.4426	0.082*	0.573 (12)
C24	0.7078 (4)	0.4224 (6)	0.4403 (2)	0.0715 (14)	
H24	0.6962	0.5056	0.4531	0.086*	
C25	0.4943 (4)	0.2442 (5)	0.6716 (2)	0.0622 (11)	
H25A	0.5009	0.3069	0.6378	0.075*	
H25B	0.5317	0.2808	0.7224	0.075*	
C26	0.8104 (3)	0.2404 (3)	0.72085 (17)	0.0358 (7)	
H26A	0.7600	0.2464	0.7454	0.043*	
H26B	0.8124	0.3249	0.7005	0.043*	
C27	0.5609 (3)	0.3046 (3)	0.52156 (17)	0.0356 (7)	
C28	0.5195 (3)	0.3363 (3)	0.43859 (18)	0.0391 (7)	
H28A	0.4547	0.2782	0.4056	0.047*	
H28B	0.4892	0.4242	0.4273	0.047*	
C29	0.5840 (4)	0.0350 (5)	0.7340 (2)	0.0641 (12)	
H29A	0.6264	−0.0379	0.7283	0.096*	
H29B	0.5093	0.0066	0.7320	0.096*	
H29C	0.6344	0.0758	0.7821	0.096*	
C30	0.8230 (3)	0.1331 (4)	0.61473 (19)	0.0427 (8)	
H30A	0.7832	0.0701	0.5753	0.064*	
H30B	0.9053	0.1062	0.6480	0.064*	
H30C	0.8238	0.2149	0.5924	0.064*	
H9A	0.168 (8)	0.191 (6)	0.713 (4)	0.051*	0.427 (12)
H21A	0.822 (10)	0.137 (10)	0.401 (4)	0.051*	0.427 (12)
H23A	0.861 (5)	0.514 (3)	0.446 (4)	0.051*	0.427 (12)

### Atomic displacement parameters (Å^2^)

**Table:**

	*U*^11^	*U*^22^	*U*^33^	*U*^12^	*U*^13^	*U*^23^
P1	0.0263 (4)	0.0470 (5)	0.0224 (4)	−0.0029 (3)	0.0044 (3)	0.0020 (3)
O2	0.0433 (13)	0.0463 (14)	0.0315 (12)	−0.0076 (11)	−0.0041 (10)	0.0055 (10)
O3	0.0459 (13)	0.0424 (13)	0.0314 (12)	0.0012 (10)	0.0103 (10)	−0.0030 (10)
N4	0.0264 (12)	0.0477 (16)	0.0241 (12)	−0.0019 (11)	0.0071 (10)	−0.0054 (11)
N5	0.0267 (12)	0.0445 (15)	0.0233 (12)	−0.0008 (11)	0.0078 (10)	−0.0001 (11)
N6	0.0292 (13)	0.077 (2)	0.0259 (13)	−0.0014 (13)	0.0098 (11)	0.0075 (13)
C7	0.054 (2)	0.088 (3)	0.052 (2)	0.017 (2)	0.0293 (19)	0.012 (2)
C8	0.057 (3)	0.169 (5)	0.054 (3)	0.016 (3)	0.033 (2)	−0.009 (3)
C9A	0.057 (3)	0.169 (5)	0.054 (3)	0.016 (3)	0.033 (2)	−0.009 (3)
C9B	0.057 (3)	0.169 (5)	0.054 (3)	0.016 (3)	0.033 (2)	−0.009 (3)
C10	0.038 (2)	0.178 (6)	0.062 (3)	0.020 (3)	0.025 (2)	0.006 (3)
C11	0.038 (2)	0.167 (6)	0.056 (3)	0.011 (3)	0.021 (2)	0.001 (3)
C12	0.043 (2)	0.155 (6)	0.054 (3)	0.007 (3)	0.024 (2)	−0.009 (3)
C13	0.0286 (15)	0.0447 (18)	0.0227 (14)	0.0003 (13)	0.0097 (12)	−0.0038 (12)
C14	0.0361 (16)	0.0420 (18)	0.0328 (16)	−0.0027 (14)	0.0128 (13)	−0.0052 (14)
C15	0.0473 (19)	0.047 (2)	0.0336 (17)	0.0094 (16)	0.0107 (15)	−0.0002 (15)
C16	0.0313 (16)	0.072 (3)	0.0304 (17)	0.0061 (16)	0.0049 (13)	−0.0038 (17)
C17	0.0321 (17)	0.072 (3)	0.0333 (17)	−0.0121 (17)	0.0064 (14)	−0.0045 (17)
C18	0.0352 (16)	0.0476 (19)	0.0300 (15)	−0.0057 (14)	0.0094 (13)	0.0010 (14)
C19	0.0342 (16)	0.071 (2)	0.0194 (14)	0.0019 (16)	0.0068 (12)	0.0086 (15)
C20	0.062 (2)	0.092 (3)	0.0348 (18)	0.027 (2)	0.0248 (18)	0.016 (2)
C21A	0.038 (6)	0.087 (8)	0.039 (4)	0.008 (4)	0.020 (4)	0.011 (5)
C21B	0.038 (6)	0.087 (8)	0.039 (4)	0.008 (4)	0.020 (4)	0.011 (5)
C22A	0.031 (5)	0.136 (15)	0.031 (5)	−0.008 (7)	0.009 (4)	0.021 (6)
C22B	0.031 (5)	0.136 (15)	0.031 (5)	−0.008 (7)	0.009 (4)	0.021 (6)
C23A	0.053 (3)	0.106 (12)	0.037 (4)	−0.038 (9)	0.015 (3)	0.002 (8)
C23B	0.053 (3)	0.106 (12)	0.037 (4)	−0.038 (9)	0.015 (3)	0.002 (8)
C24	0.064 (3)	0.112 (4)	0.0321 (19)	−0.030 (3)	0.0173 (18)	0.005 (2)
C25	0.064 (3)	0.085 (3)	0.043 (2)	0.018 (2)	0.0297 (19)	0.009 (2)
C26	0.0300 (15)	0.0428 (18)	0.0286 (15)	−0.0003 (13)	0.0093 (12)	−0.0031 (13)
C27	0.0288 (14)	0.0449 (18)	0.0291 (15)	0.0055 (13)	0.0106 (12)	0.0005 (14)
C28	0.0340 (16)	0.0467 (19)	0.0286 (15)	0.0033 (14)	0.0086 (12)	0.0049 (14)
C29	0.066 (3)	0.081 (3)	0.042 (2)	−0.004 (2)	0.0234 (19)	0.007 (2)
C30	0.0329 (16)	0.062 (2)	0.0305 (16)	−0.0016 (15)	0.0134 (13)	−0.0055 (15)

### Geometric parameters (Å, °)

**Table:**

P1—O2	1.484 (3)	C17—C18	1.391 (5)
P1—N6	1.621 (3)	C17—H17	0.9300
P1—N4	1.633 (3)	C18—H18	0.9300
P1—N5	1.681 (3)	C19—C24	1.380 (6)
O3—C27	1.221 (4)	C19—C20	1.391 (6)
N4—C30	1.466 (4)	C19—C28	1.502 (5)
N4—C26	1.477 (4)	C20—C21B	1.307 (12)
N5—C27	1.371 (4)	C20—H20	0.9300
N5—H5	0.8600	C21A—C22A	1.373 (14)
N6—C25	1.425 (5)	C21A—H21A	0.97 (11)
N6—C29	1.482 (5)	C21B—C22B	1.387 (14)
C7—C12	1.371 (6)	C21B—H21B	0.9300
C7—C8	1.389 (6)	C21B—H21A	0.95 (11)
C7—C25	1.552 (6)	C22A—C23A	1.380 (17)
C8—C9A	1.30 (2)	C22A—H22A	0.9300
C8—C9B	1.498 (18)	C22B—C23B	1.38 (2)
C8—H8	0.9300	C22B—H22B	0.9300
C9A—H9A	0.94 (2)	C22B—H21A	1.27 (11)
C9B—C10	1.254 (14)	C23A—H23A	0.92 (2)
C9B—H9B	0.9300	C23B—C24	1.470 (19)
C9B—H9A	1.05 (5)	C23B—H23B	0.9300
C10—C11	1.375 (7)	C24—H24	0.9300
C10—H10	0.9300	C25—H25A	0.9700
C11—C12	1.382 (6)	C25—H25B	0.9700
C11—H11	0.9300	C26—H26A	0.9700
C12—H12	0.9300	C26—H26B	0.9700
C13—C18	1.382 (5)	C27—C28	1.522 (4)
C13—C14	1.387 (5)	C28—H28A	0.9700
C13—C26	1.508 (4)	C28—H28B	0.9700
C14—C15	1.385 (5)	C29—H29A	0.9600
C14—H14	0.9300	C29—H29B	0.9600
C15—C16	1.376 (6)	C29—H29C	0.9600
C15—H15	0.9300	C30—H30A	0.9600
C16—C17	1.378 (6)	C30—H30B	0.9600
C16—H16	0.9300	C30—H30C	0.9600
			
O2—P1—N6	112.48 (17)	C21B—C20—H20	114.6
O2—P1—N4	111.09 (15)	C19—C20—H20	114.6
N6—P1—N4	109.09 (14)	C22A—C21A—H21A	114 (7)
O2—P1—N5	106.38 (13)	C20—C21B—C22B	112.9 (12)
N6—P1—N5	109.18 (14)	C20—C21B—H21B	123.6
N4—P1—N5	108.50 (14)	C22B—C21B—H21B	123.6
C30—N4—C26	114.1 (3)	C20—C21B—H21A	166 (5)
C30—N4—P1	117.0 (2)	C22B—C21B—H21A	63 (7)
C26—N4—P1	125.0 (2)	H21B—C21B—H21A	62.5
C27—N5—P1	126.3 (2)	C21A—C22A—C23A	123.2 (15)
C27—N5—H5	116.9	C21A—C22A—H22A	118.4
P1—N5—H5	116.9	C23A—C22A—H22A	118.4
C25—N6—C29	117.4 (3)	C23B—C22B—C21B	120.1 (12)
C25—N6—P1	125.7 (3)	C23B—C22B—H22B	120.0
C29—N6—P1	116.5 (3)	C21B—C22B—H22B	120.0
C12—C7—C8	117.2 (4)	C23B—C22B—H21A	159 (5)
C12—C7—C25	120.6 (4)	C21B—C22B—H21A	42 (5)
C8—C7—C25	122.2 (4)	H22B—C22B—H21A	79.3
C9A—C8—C7	126.0 (10)	C22A—C23A—H23A	121 (3)
C9A—C8—C9B	8.3 (14)	C22B—C23B—C24	126.0 (9)
C7—C8—C9B	118.4 (7)	C22B—C23B—H23B	117.0
C9A—C8—H8	117.0	C24—C23B—H23B	117.0
C7—C8—H8	117.0	C22B—C23B—H23A	144 (3)
C9B—C8—H8	124.5	C24—C23B—H23A	90 (2)
C8—C9A—H9A	124 (6)	H23B—C23B—H23A	27.8
C10—C9B—C8	121.2 (14)	C19—C24—C23B	110.2 (7)
C10—C9B—H9B	119.4	C19—C24—H24	124.9
C8—C9B—H9B	119.4	C23B—C24—H24	124.9
C10—C9B—H9A	136 (6)	N6—C25—C7	109.8 (4)
C8—C9B—H9A	101 (5)	N6—C25—H25A	109.7
H9B—C9B—H9A	22.8	C7—C25—H25A	109.7
C9B—C10—C11	119.8 (10)	N6—C25—H25B	109.7
C9B—C10—H10	120.1	C7—C25—H25B	109.7
C11—C10—H10	120.1	H25A—C25—H25B	108.2
C10—C11—C12	121.8 (5)	N4—C26—C13	110.8 (3)
C10—C11—H11	119.1	N4—C26—H26A	109.5
C12—C11—H11	119.1	C13—C26—H26A	109.5
C7—C12—C11	121.1 (4)	N4—C26—H26B	109.5
C7—C12—H12	119.4	C13—C26—H26B	109.5
C11—C12—H12	119.4	H26A—C26—H26B	108.1
C18—C13—C14	118.7 (3)	O3—C27—N5	122.7 (3)
C18—C13—C26	120.3 (3)	O3—C27—C28	122.1 (3)
C14—C13—C26	121.0 (3)	N5—C27—C28	115.1 (3)
C15—C14—C13	120.2 (3)	C19—C28—C27	107.2 (2)
C15—C14—H14	119.9	C19—C28—H28A	110.3
C13—C14—H14	119.9	C27—C28—H28A	110.3
C16—C15—C14	120.8 (4)	C19—C28—H28B	110.3
C16—C15—H15	119.6	C27—C28—H28B	110.3
C14—C15—H15	119.6	H28A—C28—H28B	108.5
C15—C16—C17	119.5 (3)	N6—C29—H29A	109.5
C15—C16—H16	120.2	N6—C29—H29B	109.5
C17—C16—H16	120.2	H29A—C29—H29B	109.5
C16—C17—C18	119.8 (3)	N6—C29—H29C	109.5
C16—C17—H17	120.1	H29A—C29—H29C	109.5
C18—C17—H17	120.1	H29B—C29—H29C	109.5
C13—C18—C17	120.9 (3)	N4—C30—H30A	109.5
C13—C18—H18	119.5	N4—C30—H30B	109.5
C17—C18—H18	119.5	H30A—C30—H30B	109.5
C24—C19—C20	119.9 (4)	N4—C30—H30C	109.5
C24—C19—C28	120.2 (4)	H30A—C30—H30C	109.5
C20—C19—C28	119.8 (4)	H30B—C30—H30C	109.5
C21B—C20—C19	130.8 (8)		
			
O2—P1—N4—C30	59.1 (3)	C13—C14—C15—C16	−0.4 (5)
N6—P1—N4—C30	−176.4 (3)	C14—C15—C16—C17	0.9 (6)
N5—P1—N4—C30	−57.5 (3)	C15—C16—C17—C18	−0.5 (6)
O2—P1—N4—C26	−144.4 (3)	C14—C13—C18—C17	1.1 (5)
N6—P1—N4—C26	−19.8 (3)	C26—C13—C18—C17	−178.7 (3)
N5—P1—N4—C26	99.0 (3)	C16—C17—C18—C13	−0.6 (5)
O2—P1—N5—C27	−151.0 (3)	C24—C19—C20—C21B	−1.3 (8)
N6—P1—N5—C27	87.4 (3)	C28—C19—C20—C21B	−176.8 (7)
N4—P1—N5—C27	−31.4 (3)	C19—C20—C21B—C22B	2.5 (13)
O2—P1—N6—C25	−145.4 (3)	C20—C21B—C22B—C23B	−0.8 (15)
N4—P1—N6—C25	90.9 (3)	C21B—C22B—C23B—C24	−2(2)
N5—P1—N6—C25	−27.6 (4)	C20—C19—C24—C23B	−1.3 (8)
O2—P1—N6—C29	42.9 (3)	C28—C19—C24—C23B	174.1 (7)
N4—P1—N6—C29	−80.8 (3)	C22B—C23B—C24—C19	2.8 (16)
N5—P1—N6—C29	160.8 (3)	C29—N6—C25—C7	−74.8 (4)
C12—C7—C8—C9A	−4.4 (11)	P1—N6—C25—C7	113.6 (3)
C25—C7—C8—C9A	177.3 (11)	C12—C7—C25—N6	−74.8 (6)
C12—C7—C8—C9B	−0.4 (13)	C8—C7—C25—N6	103.4 (6)
C25—C7—C8—C9B	−178.7 (9)	C30—N4—C26—C13	−61.7 (4)
C9A—C8—C9B—C10	151 (12)	P1—N4—C26—C13	141.1 (2)
C7—C8—C9B—C10	−6(2)	C18—C13—C26—N4	128.4 (3)
C8—C9B—C10—C11	8(2)	C14—C13—C26—N4	−51.4 (4)
C9B—C10—C11—C12	−5.1 (15)	P1—N5—C27—O3	−22.2 (4)
C8—C7—C12—C11	3.3 (9)	P1—N5—C27—C28	154.3 (2)
C25—C7—C12—C11	−178.4 (6)	C24—C19—C28—C27	−87.2 (4)
C10—C11—C12—C7	−0.9 (11)	C20—C19—C28—C27	88.2 (4)
C18—C13—C14—C15	−0.6 (5)	O3—C27—C28—C19	79.8 (4)
C26—C13—C14—C15	179.2 (3)	N5—C27—C28—C19	−96.7 (3)

### Hydrogen-bond geometry (Å, °)

**Table:**

*D*—H···*A*	*D*—H	H···*A*	*D*···*A*	*D*—H···*A*
C25—H25A···O3	0.97	2.49	3.347 (5)	147.
N5—H5···O2^i^	0.86	1.95	2.763 (3)	156.
C28—H28A···O2^i^	0.97	2.57	3.351 (4)	138.
C17—H17···O2^ii^	0.93	2.51	3.443 (5)	176.
C28—H28B···O3^iii^	0.97	2.40	3.325 (4)	160.

Symmetry codes: (i) −*x*+1, −*y*, −*z*+1; (ii) −*x*+2, *y*+1/2, −*z*+3/2; (iii) −*x*+1, −*y*+1, −*z*+1.
